# Longitudinal assessment of the health-related quality of life among older people with diabetes: results of a nationwide study in New Zealand

**DOI:** 10.1186/s12902-020-0519-4

**Published:** 2020-03-05

**Authors:** Seyed Morteza Shamshirgaran, Christine Stephens, Fiona Alpass, Nayyereh Aminisani

**Affiliations:** 1 Healthy Ageing Research Centre, Neyshabur University of Medical Sciences, Neyshabur, Iran; 2 Epidemiology and Biostatistics Department, Neyshabur University of Medical Sciences, Neyshabur, Iran; 3 School of Psychology, Massey University, Palmerston North, New Zealand

**Keywords:** Older adults, Diabetes mellitus, Quality of life, Longitudinal study, Community-dwelling

## Abstract

**Background:**

The current work examined experiences of Health-Related Quality of Life (HRQOL) among older adults with a diagnosis of Diabetes Mellitus (DM) over time compared to those without a diagnoses DM.

**Methods:**

The sample was drawn from six biennial waves of the New Zealand Health, Work and Retirement survey, a prospective population-based cohort study of older adults 55–70 years at baseline. Data on sociodemographic factors, health behaviours, chronic disease diagnoses and physical and mental HRQOL (SF-12v2) were obtained using six biennial surveys administered 2006–2016. Generalised Estimating Equation models, adjusted for time-constant and -varying factors, were employed to compare HRQOL and its determinants over time for older adults with and without a diagnosis of DM.

**Results:**

DM was negatively associated with physical HRQOL [β (95% CI) − 7.43 (− 8.41, − 6.44)] with older adults affected by DM reporting scores 7.4 points lower than those without DM. Similarly, the mean Mental HRQOL score was lower among those affected by DM [β = − 4.97 (− 5.93, − 4.01)] however, scores increased over time for both groups (*p* < 0.001). Greater age, more chronic conditions, sight and sleep problems, obesity, lower annual income, and fewer years of education were predictors of poorer HRQOL among older adults.

**Conclusions:**

Older adults affected by diabetes experienced poorer physical and mental HRQOL compared to those not affected when controlling for a range of sociodemographic and health related indices. A management aim must be to minimise the gap between two groups, particularly as people age.

## Background

Diabetes Mellitus (DM) is a significant health concern worldwide that is increasing in many regions [1]. According to the International Federation of Diabetes, there were 415 million adults with diabetes around the globe in 2015, and this figure increased in the following years [2]. Similarly, in New Zealand, the prevalence of DM has been steadily growing over the last 30 years [3, 4] with Māori and Pacific populaitions both disproportionally affected [5] and experaince higher diabetes-related mortality in later life [4]. Indeed, Type 2 diabetes is an important consideration for older adults, who are at increased risk in terms of onset and disease management due to age-related changes in metabolism and who represent a considerable number of patients [6]. Indeed, disease-related complications, poorly controlled cardiovascular risk, impaired cognitive function, loss of mobility, increased dependency and service use are more common in older adults with diabetes [6].

Quality of Life (QOL), defined as “individual’s perception of their position in life in the context of the culture and value systems in which they live and in relation to their goals, expectations, standards, and concerns” [7], is an important consideration in chronic disease management [8]. Health-Related Quality of Life (HRQOL) refers more specifically to these perceptions as they pertain to subjective impacts of physical, mental and social health states [9] and it is well recognised that the severe short and long-term complications arising from DM [10, 11] can have a negative impact on HRQOL [12]. Previous studies have demonstrated that older adults with diabetes report poorer physical HRQOL than the general population [13]. However, while cross-sectional studies showed the levels and determinants of HRQOL among those with a diagnosis of DM [14–18], such designs are unable to investigate these declines in HRQOL among adults affected by DM in older age or to clarify whether predictors may influence any such reduction.

Despite this, relatively few studies have investigated the longitudinal changes in HRQOL among people with DM. A population-based study of adults aged 18 and over in the USA followed groups with and without a baseline diagnosis of Type 2 diabetes between 2004 and 2009 [19]. Among these groups, analyses of change in EQ-5D scores over the 5 years showed that HRQOL among those with a diagnosis of DM declined at around twice the rate observed for those without DM. While respondents in this population sample included a broad age range, it is unclear whether this rate of change and determinants identified for those with and without a DM diagnosis reflect the experiences of older adults, for whom co-morbidities and other age-related changes in activity and metabolism may enhance the impacts of DM.

Two other studies of the impact of DM on HRQOL were not population-based and did not include people without DM as a comparison group [17, 20], making it difficult to separate the impacts of age from effects of DM. Bayliss et al. compared the effect of diabetes on HRQOL with other chronic diseases measured over four years [20]. Compared to other conditions, adults with comorbid diabetes displayed the most significant declines in physical HRQOL over time, suggesting that the physical impacts of diabetes are progressive even over a moderate follow-up period Results of a longitudinal study conducted in Germany using data from general practices between 2000 and 2007, showed a decline in HRQOL scores among people with DM which was more significant in the presence of diabetes-related complication and comorbidities. A higher number of diabetes related complications and a reported history of depression and lower HRQOL at baseline were predictors of decreased Mental Component Score (MCS), while complications, Body Mass Index (BMI), smoking and HRQOL at baseline significantly predicted Physical Component Score (PCS) over five years of follow-up [17]. The most recent longitudinal study (mean observation period of 8.7 years) using pooled data from 5367 people aged 45–74 in Germany found an annual decline in HRQOL scores in the DM group compared with people without DM [21].

To understand changes in HRQOL and the actual burden of diabetes among older age groups, longitudinal assessment with longer follow-up is valuable. The longitudinal data will enable researchers to examine changes in psychological and behavioural antecedents of diabetes specifically among Maori and pacific people. The objective of the current study was to assess the changes in HRQOL over ten years among people with diabetes, using data from the New Zealand Health Work and Retirement Study (HWR). We aimed to compare the QOL scores over time between groups with prevalent DM compared with people without DM based on data from 6 waves of a nationwide longitudinal study on ageing in New Zealand.

## Methods

### Study population

**Fig. 1:**
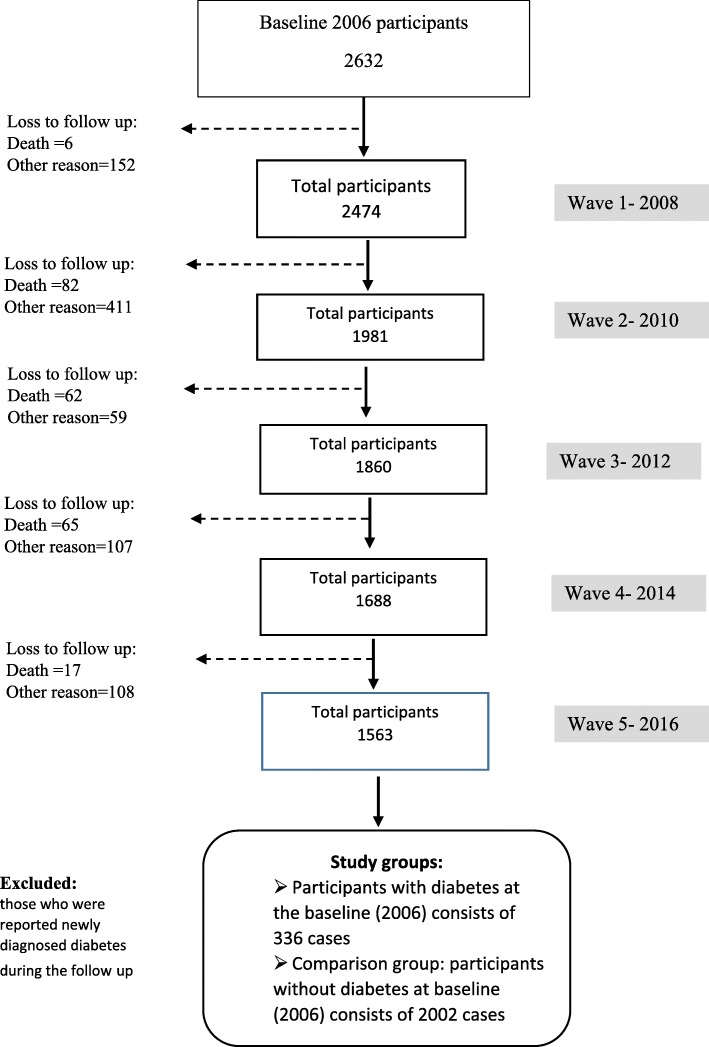
Flow Diagram of Study Participants

The sample was drawn from the 2006–2016 waves of the New Zealand HWR study, a prospective cohort study of community-dwelling older adults. The HWR commenced in 2006 as a biennial postal survey of a sample aged 55–70, randomly selected from the New Zealand electoral roll. An over-sample of adults of Māori decent was undertaken to ensure adequate representation of this section of the older population. The core questionnaire assesses domains of health and wellbeing; family and social support; work and retirement; financial wellbeing; and cultural identity. Cases included in the current analyses participated in the baseline survey and at least one subsequent follow up period and provided information on health-related quality of life at two or more time points. The original survey had a response rate of 53% (*n* = 6662), and of these, 2632 consented to participate in subsequent follow up. Compared to those who dropped out, those who completed at least one wave of follow up were older (60.8 ± 4.5 vs. 61.1 ± 4.6, *p* = 0.033), non-Māori (50.6% vs. 58.2%, *p* < 0.001), and more male (45.1% vs. 46.6%, *p* = 0.211). Of those willing to participate in longtidunal follow up, 1609 (41%) were lost to follow up over the five biennial follow up waves (212 to death, and remaining unknown). For the current analysis, participants with a diagnosis of diabetes at baseline who responded to at least one subsequent survey were selected, A comparison group of those without diabetes at the baseline who remained free from DM in all follow- up surveys (2008–2016) were selected. We excluded incident cases of diabetes (*n* = 192). Figure 1 presents a flow chart illustrating inclusion criteria and attrition 2006–2016.

### Measures

#### Sociodemographic variables

Demographic variables were as follows: age as two categories of 55–64 years and 65 and over and as a continuous variable, marital status in three groups of married/living with a partner, divorced/ separated/single, and widowed, ethnicity as Māori (indigenous New Zealanders) and non-Māori (Europeans, Asians, Pacific people and others) according to the priority ethnic groups in NZ. Education was categorised as no secondary, secondary, post-secondary and tertiary. Annual personal income was categorised as 0–25,000, 25,001–50,000, 50,001–70,000 and > 70,000 NZ$.

#### Clinical variables

A list of self-reported doctor-diagnosed physical and mental conditions for the current analysis includes cardiovascular diseases (Heart conditions, stroke), neurologic diseases (epilepsy, Alzheimer/dementia, Parkinson, migraine headache), musculoskeletal (arthritis, osteoporosis, hip/knee replacement), asthma and Chronic Obstructive Pulmonary Diseases (COPD)), chronic liver diseases (cirrhosis), depression and other mental illness, and cancer. The sum of chronic conditions was calculated and categorised as none, one, two and more conditions. Hypertension, hearing and eye problems or sleep disorder, were considered separately as dichotomous variables. Height and weight were measured in the 2008 survey wave and was used to calculated BMI as healthy weight (< 25), overweight (25–29.9), and obese (≥30).

#### Health behaviours

Current smokers were those who identified themselves as a regular smoker. Alcohol consumption was classified into two categories: regular alcohol consumption (2 or more drinks per week), and non-regular alcohol drinkers (≤1 drink per week). Physical activity (moderate/brisk walking or vigorous activity) over the last seven days were categorised into two levels: two or more times per week (sufficient), once per week/none (insufficient).

#### Health related quality of life

Physical HRQOL was assessed using the SF12v2 [22]. Ten items of the SF12v2 are rated and a scale of 1–5 and two items on a scale of 1–3. Standardised norm based orthogonal factor weights are used to calculate a PCS [positive weights for physical functioning (2 items), role physical (2 items), pain and general health] and a MCS [positive weights for vitality, social functioning, relationships (2 items) and mental health (2 items)] with reference a New Zealand population mean of 50 and standard deviaiton of 10 [23].

### Statistical analysis

Data were extensively screened. Missing data for chronic conditions such as hypertension, stroke, and so on in some waves were replaced with available data from the preceding or the subsequent waves over the study period. All possible comparisons of the age/year at diagnosis in each wave were checked to ensure that the most reliable list was utilised.

Descriptive analyses were usd to describe the characteristics of the sample. The absolute changes in PCS and MCS were calculated by subtracting the follow-up score from the baseline score, which was the first SF12 completed for each person. For those with multiple scores, the average over time was considered.

A Generalised Estimating Equation (GEE) model was performed to examine the association between demographic, clinical and health behaviours variables with and HRQOL over time to account for longitudinal within-subject correlations. Variables were introduced as fixed (sex, ethnicity, education, personal income, BMI) or time-varying (other variables) into the models based on availability and completeness of the data in each wave. Two models were fitted separately for the physical and mental dimensions of the SF12; the comparison group for each model was people without diabetes. As a sensitivity analysis, the difference between SF12 scores for DM and non-DM participants was also estimated for its baseline values in both crude and adjusted models. Data were analysed using the STATA statistical package version 14; all estimates were reported with 95% confidence interval and a significance level of 0.05.

## Results

Table 1 describes the range of epidemiological and clinical variables and PCS and MCS. Relative to non-DM, DM participants were older (61.6 ± 4.4 vs 60.9 ± 4.5 respectively, *p* = 0.016), less educated, less well off (*p* < 0.001), and more likely to identify as Māori, (*p* = 0.002). Rates of current smoking, overweight/obesity, irregular alcohol consumption, insufficient physical activity (less than two times moderate/vigorous activity per week), were higher among DM participants compared to non-DM participants (*p* < 0.001). A number of medical conditions, hypertension, sight, hearing and sleep problems were higher among DM participants relative to non-DM participants (p < 0.001). In general, the mean scores of both physical and mental dimensions of SF12 were significantly lower among DM participants. Within each level of sociodemographic, lifestyle and clinical factors, DM participants had poorer performance on HRQOL (Table 1).

**Table 1:** Baseline health related quality of life, clinical and epidemiological characteristics of people with/without diabetes

Characteristics	Diabetes		SF-12-PCS Mean ± SD		SF-12-MCS Mean ± SD	
	with diabetes	without diabetes	with diabetes	without diabetes	with diabetes	without diabetes
**Age Groups**						
55–64	240 (71.4)	1490 (74.4)	41.3 ± 10.8	49.3 ± 8.7	44.8 ± 12.4	49.8 ± 10.1
64+	96 (28.6)	512 (25.6)	38.9 ± 11.5	45.7 ± 10.1	45.0 ± 12.3	51.3 ± 9.7
**Sex**						
Male	169 (50.3)	923 (46.1)	42.0 ± 10.9	48.9 ± 8.7	45.4 ± 12.1	50.6 ± 9.6
Female	167 (49.7)	1079 (53.9)	39.3 ± 11.0	47.9 ± 9.6	44.3 ± 12.7	49.9 ± 10.4
**Education**						
No secondary	128 (38.8)	536 (27.0)	40.4 ± 10.6	46.4 ± 9.7	43.8 ± 11.1	49.2 ± 11.1
Secondary	79 (23.9)	557 (28.0)	39.9 ± 11.8	48.5 ± 9.3	46.2 ± 12.5	50.0 ± 10.1
Post- secondary/tertiary	123 (37.3)	895 (45)	41.5 ± 11.1	49.6 ± 8.6	45.3 ± 13.5	50.9 ± 9.3
**Marital Status**						
Married/partner	218 (65.9)	1473 (74.6)	41.7 ± 10.3	48.9 ± 8.8	46.6 ± 11.1	50.8 ± 9.4
Divorced/separated/single	72 (21.8)	365 (18.5)	38.6 ± 11.6	47.3 ± 10.1	41.4 ± 13.5	48.4 ± 11.2
Widowed	41 (12.4)	137 (6.9)	38.0 ± 12.4	46.2 ± 9.9	41.6 ± 15.1	48.2 ± 11.8
**Annual Personal Income**						
0–25,000	139 (54.9)	659 (38.6)	38.6 ± 11.2	46.0 ± 10.3	44.9 ± 12.8	48.6 ± 11.3
25,001–50,000	80 (31.6)	596 (34.9)	42.8 ± 10.8	50.1 ± 7.7	45.3 ± 12.0	51.2 ± 8.9
50,001–70,000	24 (9.5)	243 (14.2)	47.1 ± 7.2	50.6 ± 7.7	48.7 ± 10.5	51.6 ± 8.2
> 70,000	10 (4.0)	211 (12.4)	48.5 ± 11.5	52.2 ± 5.8	44.2 ± 15.4	51.9 ± 7.7
**Ethnicity**						
European/others	114 (34.6)	1242 (63.1)	40.6 ± 11.0	48.9 ± 9.0	46.1 ± 12.0	50.5 ± 9.6
Maori	216 (65.5)	725 (35.9)	40.5 ± 11.1	47.5 ± 9.5	44.0 ± 12.6	49.4 ± 10.8
**Current Smoker (Yes)**	66 (20.4)	255 (13.1)	40.8 ± 11.1	47.1 ± 10.3	41.1 ± 13.4	47.6 ± 12.0
**Regular Alcohol Consumption (Yes)**	67 (20.0)	1015 (51.0)	44.4 ± 9.9	49.6 ± 8.5	46.8 ± 11.8	51.2 ± 9.2
**BMI**						
< 25	41 (14.4)	584 (34.4)	41.3 ± 11.8	50.1 ± 8.2	44.4 ± 12.5	50.9 ± 9.4
25–29.9	80 (28.1)	652 (38.4)	43.9 ± 11.9	49.2 ± 8.4	46.8 ± 12.2	50.7 ± 9.6
≥ 30	164 (57.5)	463 (27.3)	39.510.3	45.4 ± 10.4	44.3 ± 12.6	48.6 ± 10.9
**Physical Activity**						
No	45 (13.9)	163 (8.4)	34.0 ± 12.2	42.4 ± 13.0	37.7 ± 14	46.2 ± 13.2
Once a week	20 (6.2)	86 (4.4)	43.4 ± 11.1	48.4 ± 8.2	43.9 ± 12.1	48.9 ± 10.2
≥ 2 times per week	260 (80.0)	1699 (87.2)	41.8 ± 10.4	49.0 ± 8.5	46.4 ± 11.7	50.7 ± 9.5
**Medical Conditions**						
None	75 (22.3)	679 (33.9)	47.2 ± 8.8	52.3 ± 6.0	48.8 ± 10.6	52.8 ± 8.3
1	96 (28.6)	737 (36.8)	43.1 ± 9.4	48.4 ± 8.6	49.0 ± 10.1	50.4 ± 9.7
≥ 2	165 (49.1)	586 (29.3)	36.5 ± 11.0	43.8 ± 10.7	40.8 ± 13.0	46.9 ± 11.2
**Hypertension (Yes)**	231 (70.9)	739 (37.1)	39.3 ± 10.9	46.4 ± 9.8	43.8 ± 12.7	48.8 ± 10.5
**Sight Problem (Yes)**	58 (18.3)	197 (9.9)	38.1 ± 9.4	45.6 ± 9.6	41.6 ± 12.8	46.9 ± 11.7
**Hearing Problem (Yes)**	35 (10.4)	451 (22.6)	40.4 ± 10.4	46.8 ± 9.7	42.9 ± 12.2	48.8 ± 10.9
**Sleep Problem (Yes)**	35 (10.4)	105 (5.2)	37.4 ± 10.9	43.6 ± 11.1	36.1 ± 13.5	44.0 ± 13.0

**Fig. 2:**
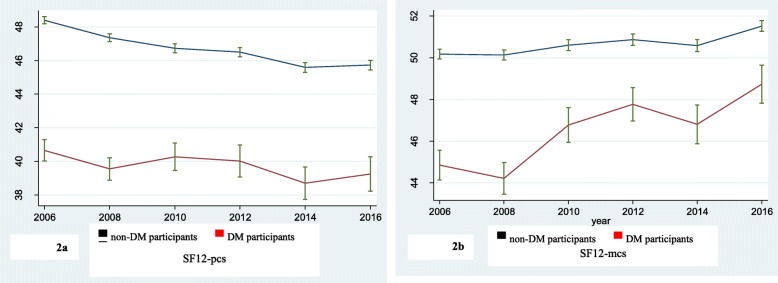
Changes in physical and mental dimensions of SF12, 2006–2016. **a** mean SF-PCS by wave in people with/without DM. **b** mean SF12-MCS by wave in people with/without DM

Figure 2 shows that the mean PCS was significantly lower for the DM participants (*p* < .001) and remained lower across time (*p* < 0.001). Results of the linear regression model showed that the PCS score decreased over time in both DM and non-DM participants, however, with greater variability among DM participants. Similarly, mean MCS was significantly lower for the DM participants and remained lower across time (*p* < 0.001). In contrast, the MCS score increased over time for the DM group, while it remained stable among non-DM participants (*p* < 0.02) (Fig. 2; Table 2).

**Table 2:** Quality of life score (two components) over time among people with/without diabetes

Outcome time	SF 12-PCS (Mean ± SD)			SF 12-MCS (Mean ± SD)		
	With diabetes	Without diabetes	***P*** value	With diabetes	Without diabetes	***P*** value
2006	40.6 ± 11.0	48.4 ± 9.2	< 0.001	44.9 ± 12.4	50.2 ± 10.0	< 0.001
2008	39.5 ± 11.1	47.4 ± 9.4	< 0.001	44.2 ± 12.6	50.1 ± 9.9	< 0.001
2010	40.3 ± 11.2	46.7 ± 10.0	< 0.001	46.8 ± 11.3	50.6 ± 9.7	< 0.001
2012	40.0 ± 12.2	46.5 ± 10.0	< 0.001	47.8 ± 10.2	50.9 ± 9.9	0.0002
2014	38.7 ± 11.3	45.6 ± 9.7	< 0.001	46.8 ± 10.9	50.6 ± 9.9	< 0.001
2016	39.2 ± 11.6	45.7 ± 9.9	< 0.001	48.7 ± 10.3	51.5 ± 9.1	0.0012
Mean follow-up SF12 score	39.1 ± 10.5	46.1 ± 8.8	< 0.001	45.2 ± 10.6	50.1 ± 8.6	< 0.001

GEE modelling demonstrated that the PCS decreased over time (*p* < 0.001), however, and there was a significant difference in the rate of change between DM and non-DM participants. Having diabetes was negatively associated with PCS [β (95% CI) − 7.43 (− 8.41, − 6.44)] indicating that individuals with DM had an average score 7.4 points lower on the PCS than those without diabetes. Although the results showed an increase in MCS (*p* < 0.001) over time, the mean of SF12-MCS was lower among DM participants compared to non-DM participants [β = − 4.97 (− 5.93, − 4.01)]. After adjustment for all other variables the effect size decreased but the mean difference remained for both PCS and MCS scores: β = − 3.51 (− 4.55, − 2.45) and β = − 1.55 (− 2.67, − 0.42) respectively. Additionally, age, having one/two and more medical conditions, sight and sleep problems, hypertension, being obese (BMI ≥ 30), higher annual income, and years of education were significant predictors of PCS and MCS (Table 3).

**Table 3:** Results of the multivariate generalized estimating equation analysis regarding association between different factors with HRQOL dimensions

Characteristics	SF12-PCS			SF12-MCS		
	β^**a**^ (SE)	95% CI	***P*** value	β^**a**^ (SE)	95% CI	***P*** value
**Diabetic**	−3.50 (0.53)	−4.55, − 2.45	0.000	−1.55 (0.57)	−2.67, − 0.42	0.007
**Age**	−0.09 (0.02)	− 0.14, − 0.05	0.000	0.27 (0.02)	0.22, 0.31	0.000
**Sex (ref: female)**	−0.54 (0.35)	−1.24, 0.15	0.130	−0.94 (0.38)	−1.69, − 0.19	0.014
**Education (ref: No Secondary)**						
Secondary	0.24 (0.46)	−0.66, 1.16	0.594	−0.86 (0.50)	−1.83, 0.12	0.087
Post- secondary/tertiary	0.92 (0.41)	0.11, 1.72	0.025	−0.17 (0.44)	−1.04, 0.68	0.684
**Marital status (ref: Married/Partner)**						
Divorced/separated/single	0.07 (0.35)	− 062, 0.77	0.832	− 1.32 (0.39)	−2.09, − 0.55	0.001
Widowed	−0.47 (0.42)	− 1.30, 0.35	0.266	−1.33 (0.47)	−2.25, − 0.41	0.005
**Annual Personal Income (0–25,000)**						
25,001–50,000	2.52 (0.39)	1.75, 3.29	0.000	2.15 (0.42)	1.33, 2.98	0.000
50,001–70,000	3.71 (0.53)	2.66, 4.75	0.000	2.80 (0.56)	1.68, 3.92	0.000
> 70,000	4.10 (0.58)	2.96, 5.25	0.000	2.77 (0.62)	1.54, 3.99	0.000
**Ethnicity (ref: European)**						
Māori	0.29 (0.35)	−0.39, 0.98	0.397	0.01 (0.37)	0.72, 0.75	0.064
**Current Smoker (ref: Non-smokers)**	−0.76 (0.40)	−1.55, 0.02	0.059	−1.73 (0.44)	−2.61, −0.86	0.000
**Regular Alcohol Consumption**^**b**^ **(ref: < 2 per weeks)**	0.79 (0.21)	0.37, 1.22	0.000	0.82 (0.24)	0.35, 1.29	0.001
**BMI (ref: < 25)**						
25–29.9	−0.65 (0.39)	−1.43, 0.12	0.098	−0.24 (0.42)	−0.59, 1.07	0.572
≥ 30	−3.66 (0.44)	−4.53, −2.79	0.000	−0.90 (0.47)	−1.83, 0.02	0.056
**Physical Activity (ref: No)**						
Once a week	2.54 (0.31)	1.93, 3.16	0.000	1.20 (0.35)	0.50, 1.89	0.001
≥ 2 times per week ^c^	3.16 (0.26)	2.65, 3.68	0.000	2.23 (0.29)	1.65, 2.80	0.000
**Medical conditions**^**d**^ **(ref: No)**						
1	−2.98 (0.32)	−3.61, − 2.35	0.000	−1.52 (0.35)	− 2.22, −0.83	0.000
2	−5.53 (0.36)	−6.26, −4.81	0.000	−3.92 (0.40)	−4.71, − 3.13	0.000
**Hypertension (ref: no)**	−1.17 (0.28)	−1.72, −0.62	0.000	−0.83 (0.30)	− 1.43, − 0.23	0.007
**Sight problem (ref: no)**	− 1.33 (0.31)	−1.95, − 0.70	0.000	−1.18 (0.35)	− 1.87, − 0.48	0.001
**Hearing problem (ref: no)**	− 0.48 (0.31)	−1.10, 0.12	0.121	−0.69 (0.34)	−1.36, − 0.02	0.042
**Sleep problem (ref: no)**	−2.17 (0.38)	− 2.93, − 1.41	0.000	−2.80 (0.42)	−3.64, − 1.96	0.000

^a^Adjusted model, ^b^ 2 and more drink per week, ^c^ 2 and more times per week brisk walking, moderate and vigorous physical activity, ^d^Medical conditions including heart, stroke, chronic respiratory including asthma, liver (cirrhosis), neurologic (epilepsy, migraine, multiple sclerosis, Parkinson, Alzheimer, dementia, mental disorders including depression)

Results of the sensitivity analysis showed that adjustment for the baseline SF-12 confirmed a decrease in the PCS and an increase in the MCS, and there was a significant difference between DM and non-DM participants. DM participants demonstrated lower QOL scores compared to non-DM participants (Table 4).

**Table 4:** Differences between SF12 scores between DM and non-DM participants adjusted for the baseline values

SF12-PCS	β (SE)	95% CI	***P*** value	SF12-MCS	β (SE)	95% CI	***P*** value
Baseline	0.66 (0.02)	0.63–0.69	< 0.001	Baseline	0.51 (0.01)	0.48–0.54	< 0.001
time	−0.30 (0.03)	−.035, −.025	< 0.001	time	0.09 (0.03)	0.03–0.14	0.003
group	−2.60 (0.44)	−3.46, −1.74	< 0.001	group	−1.77 (0.44)	− 2.63-0.90	< 0.001

## Discussion

The current study examined the longitudinal changes in HRQOL among older people with DM and without DM over ten years of follow-up. The results showed that the SF12-PCS decreased over time and having diabetes was negatively associated with the HRQOL-physical dimension. Although the results showed an increase in SF12-MCS over time, the mean of SF12-MCS was lower among DM participants compared to non-DM participants. Grandy et al. [19] in their longitudinal study found the decline in the EQ-5D index score people with diabetes about twice the size of the decline in people without diabetes over 5 years. In a recent German study by Schunk et al. [21] using a longitudinal approach over a mean follow-up of 8.7 years, the annual decline in HRQOL scores (both MCS and PCS) in the group with prevalent diabetes was significantly larger than in people without diabetes. Our results found no difference in the decline of the HRQOL scores, but the difference between groups remained significant over time. This indicates the people with DM have poorer performance in both dimensions of the HRQOL at starting point compared to those without DM from the baseline, and this difference remained significant over time. Similar to cross-sectional studies we found that the SF12-PCS are lower among those with DM compared to people without DM [24].

There are two possible reasons for the differences between our results and the previous studies: instruments and measurement points. We used the SF12 instrument to assess QOL while Grandy et al. [19] used the EQ-5D index which has more ceiling effect and less breadth of mental dimensions compared to the SF12. The difference between our study and the German study [21] is the number of the study waves; compared to only two measurement points, the five measurement points in our study provides greater stability in mapping change over time.

We also found that age, having one/two and more medical conditions, sight and sleep problems, hypertension, being obese (BMI ≥ 30), higher annual income, and years of education were significant predictors of HRQOL. Maatuk et al. [17] in a longitudinal study found age, smoking, diabetes complications, and BMI associated with PCS decline and for a decrease in the MCS, diabetes complications and self-report depression were significant. We found that those with DM who were living alone had poorer MCS over time compared to those without DM. However, a recent German study found a reverse effect which suggests the need for more research about the living arrangement and social support [25].

Like Schunk et al. [21] we did not find significant gender differences for PCS however, in contrast, in our study women had lower MCS scores than men. Cross-sectional studies have also shown a more pronounced effect of diabetes on MCS [25, 26].

Despite the value of longitudinal nature of the current study to assess variation in the HRQOL among DM and non-DM participants over five follow up waves using a national sample of New Zealanders, we acknowledge that results might be vulnerable to biases. First of all generalizability of our sample should be considered with caution because of the participation rate, dropped outs, and loss to follow up. However, this is a large nationally sample of older New Zealanders which includes a large subsample of Maori people allowing different comparisons. Secondly, data collection for this longitudinal study was based on mailed surveys and using the self-report approach, this is subject to recall and reporting biases and may not truly reflect the diabetes status of participants. - As such, the magnitude of the differences between DM and non-DM groups may be lower than those observed by studies accessing clinical records. However, the validity of using self-report has been confirmed in previous studies [27], and thus current estimates it can advance our understanding of HRQOL among those with diagnosed DM in the New Zealand context. Another limitation is missing values due to death or other reasons; we used GEE modelling to address the problem however, future research may usefully employ different methods, such as multiple imputations. Interferences under the GEE method are valid with the strong assumption that the missing data are missing completely at random. When response data are missing at random, the GEE method should be modified either by inverse-probability weighting or by multiple imputations [28]. Weight and height were based on the self-report approach which is subject to recall and under/ over -reporting biases. We also considered the BMI as the fixed variable since the data was available only in wave 2008, however BMI might be changed over time for some reasons such as the weight loss following diagnosis of diabetes as a part of diabetes management, we recommend that other studies should consider measuring weight and height for BMI calculation using tools and over the study period. Finally, selection bias is a potential problem when attempting to generalise estimates obtained in this study to the broader population of older adults as those who accepted to stay in the study might be not a representative sample of the older New Zealand population and the effect size accordingly be underestimated as those with greater health-related barriers to participation may be under represented.

## Conclusions

Our study demonstrates that decline in HRQOL over time was similar for those with and without DM; however, the difference between the scores of HRQOL between DM and non-DM participants at the baseline remained significant over time. This indicates that diabetes has a continuing negative impact on HRQOL; therefore, disease management plans should seek to address this burden. An inverse association between age and HRQOL reflects that diabetes care and management should target elderly individuals to enhance the general health and well-being of older adults with diabetes.

Efforts to improve diabetes management, including evidence-based treatment and advice for self-management [29], are crucial to alleviating the diabetes burden in afflicted patients especially older adults.

## Data Availability

The data or analysis generated during this study is available from the corresponding author upon reasonable request.

## Abbreviations

BMI: Body Mass Index
COPD: Chronic Obstructive Pulmonary Diseases
DM: Diabetes Mellitus
GEE: Generalised Estimating Equation;
HRQOL: Health Related Quality of Life;
HWR: Health Work and Retirement Study
MCS: Mental Component Score
PCS: Physical Component Score

## References

[1] Danaei G, Finucane MM, Lu Y, Singh GM, Cowan MJ, Paciorek CJ (2011). National, regional, and global trends in fasting plasma glucose and diabetes prevalence since 1980: systematic analysis of health examination surveys and epidemiological studies with 370 country-years and 2.7 million participants. Lancet.

[2] Atlas ID. Brussels, Belgium: International Diabetes Federation; 2013. International Diabetes Federation (IDF). 2017.

[3] Ministry of Health (2014). Virtual diabetes register: estimated diagnosed cases of diabetes by DHB as at December 2013.

[4] Joshy G, Simmons D (2006). Epidemiology of diabetes in New Zealand: revisit to a changing landscape. N Z Med J.

[5] Ministary of Health. Annual Data Explorer 2016/17: New Zealand Health Survey [Data File]. 2017. URL: https://minhealthnz.shinyapps.io/nz-health-survey-2016-17-annual-update.

[6] Chentli F, Azzoug S, Mahgoun S (2015). Diabetes mellitus in elderly. Indian J Endocrinol Metab.

[7] Group W (1995). The World Health Organization quality of life assessment (WHOQOL): position paper from the World Health Organization. Soc Sci Med.

[8] Burckhardt CS, Anderson KL (2003). The quality of life scale (QOLS): reliability, validity, and utilization. Health Qual Life Outcomes.

[9] Hennessy CH, Moriarty DG, Zack MM, Scherr PA, Brackbill R (1994). Measuring health-related quality of life for public health surveillance. Public Health Rep.

[10] Shamshirgaran SM, Mamaghanian A, Aliasgarzadeh A, Aiminisani N, Iranparvar-Alamdari M, Ataie J (2017). Age differences in diabetes-related complications and glycemic control. BMC Endocr Disord.

[11] Solli O, Stavem K, Kristiansen IS (2010). Health-related quality of life in diabetes: the associations of complications with EQ-5D scores. Health Qual Life Outcomes.

[12] Venkataraman K, Wee HL, Leow MK, Tai ES, Lee J, Lim SC (2013). Associations between complications and health-related quality of life in individuals with diabetes. Clin Endocrinol.

[13] Wandell PE, Tovi J (2000). The quality of life of elderly diabetic patients. J Diabetes Complicat.

[14] Didarloo A, Alizadeh M (2016). Health-related quality of life and its determinants among women with diabetes mellitus: a cross-sectional analysis. Nurs Midwifery Stud.

[15] Jain V, Shivkumar S, Gupta O (2014). Health-related quality of life (hr-qol) in patients with type 2 diabetes mellitus. N Am J Med Sci.

[16] Kiadaliri AA, Najafi B, Mirmalek-Sani M (2013). Quality of life in people with diabetes: a systematic review of studies in Iran. J Diabetes Metab Disord.

[17] Maatouk I, Wild B, Wesche D, Herzog W, Raum E, Muller H (2012). Temporal predictors of health-related quality of life in elderly people with diabetes: results of a German cohort study. PLoS One.

[18] Shamshirgaran SM, Ataei J, Iranparvar Alamdari M, Safaeian A, Aminisani N (2016). Predictors of health-related quality of life among people with type II diabetes mellitus in Ardabil, northwest of Iran, 2014. Prim Care Diabetes.

[19] Grandy S, Fox KM (2012). Change in health status (EQ-5D) over 5 years among individuals with and without type 2 diabetes mellitus in the SHIELD longitudinal study. Health Qual Life Outcomes.

[20] Bayliss EA, Bayliss MS, Ware JE, Steiner JF (2004). Predicting declines in physical function in persons with multiple chronic medical conditions: what we can learn from the medical problem list. Health Qual Life Outcomes.

[21] Schunk M, Reitmeir P, Ruckert-Eheberg IM, Tamayo T, Schipf S, Meisinger C (2017). Longitudinal change in health-related quality of life in people with prevalent and incident type 2 diabetes compared to diabetes-free controls. PLoS One.

[22] Ware JE, Kosinski M, Keller SD (1996). A 12-item short-form health survey: construction of scales and preliminary tests of reliability and validity. Med Care.

[23] Ware J, Kosinski M, Turner-Bowker D, Gandek B (2002). How to score version 2 of the SF-12 health survey (with a supplement documenting version 1).

[24] Schunk M, Reitmeir P, Schipf S, Volzke H, Meisinger C, Thorand B (2012). Health-related quality of life in subjects with and without type 2 diabetes: pooled analysis of five population-based surveys in Germany. Diabetic Med.

[25] Schunk M, Reitmeir P, Schipf S, Volzke H, Meisinger C, Ladwig KH (2015). Health-related quality of life in women and men with type 2 diabetes: a comparison across treatment groups. J Diabetes Complicat.

[26] Maddigan SL, Feeny DH, Majumdar SR, Farris KB, Johnson JA (2006). Understanding the determinants of health for people with type 2 diabetes. Am J Public Health.

[27] Comino EJ, Tran DT, Haas M, Flack J, Jalaludin B, Jorm L (2013). Validating self-report of diabetes use by participants in the 45 and up study: a record linkage study. BMC Health Serv Res.

[28] Birhanu T, Molenberghs G, Sotto C, Kenward MG (2011). Doubly robust and multiple-imputation-based generalized estimating equations. J Biopharm Stat.

[29] Powers MA, Bardsley J, Cypress M, Duker P, Funnell MM, Fischl AH (2015). Diabetes self-management education and support in type 2 diabetes: a joint position statement of the American Diabetes Association, the American Association of Diabetes Educators, and the academy of nutrition and dietetics. J Acad Nutr Diet.

